# Role of Calcium Signaling in the Transcriptional Regulation of the Apicoplast Genome of *Plasmodium falciparum*

**DOI:** 10.1155/2014/869401

**Published:** 2014-04-27

**Authors:** Sabna Cheemadan, Ramya Ramadoss, Zbynek Bozdech

**Affiliations:** School of Biological Sciences, Nanyang Technological University, 60 Nanyang Drive, Singapore 637551

## Abstract

Calcium is a universal second messenger that plays an important role in regulatory processes in eukaryotic cells. To understand calcium-dependent signaling in malaria parasites, we analyzed transcriptional responses of *Plasmodium falciparum* to two calcium ionophores (A23187 and ionomycin) that cause redistribution of intracellular calcium within the cytoplasm. While ionomycin induced a specific transcriptional response defined by up- or downregulation of a narrow set of genes, A23187 caused a developmental arrest in the schizont stage. In addition, we observed a dramatic decrease of mRNA levels of the transcripts encoded by the apicoplast genome during the exposure of *P. falciparum* to both calcium ionophores. Neither of the ionophores caused any disruptions to the DNA replication or the overall apicoplast morphology. This suggests that the mRNA downregulation reflects direct inhibition of the apicoplast gene transcription. Next, we identify a nuclear encoded protein with a calcium binding domain (EF-hand) that is localized to the apicoplast. Overexpression of this protein (termed PfACBP1) in *P. falciparum* cells mediates an increased resistance to the ionophores which suggests its role in calcium-dependent signaling within the apicoplast. Our data indicate that the *P. falciparum* apicoplast requires calcium-dependent signaling that involves a novel protein PfACBP1.

## 1. Introduction

Malaria is the most deadly parasitic disease and yet it is still one of the most common infectious diseases in the tropical and subtropical region of the planet. Approximately 2 billion people (40% of the world's population) are at risk of malaria infection in over 90 countries. Each year 300–500 million cases are being reported out of which over one million cases result in death [1]. Artemisinin-based combination therapies (ACT) are presently recommended as the first line of malaria treatment and their effectiveness underline many major accomplishments of the world wide malaria control programs over the last decade. Unfortunately, there are alarming reports of reduced sensitivity to ACTs emerging in Southeast Asia that poses a major threat for the future [2–4]. Based on the experience with previously deployed antimalaria chemotherapeutics such as chloroquine (1950s) and antifolates in (1960s), a spread of artemisinin resistance around the world could erase all advances of the malaria control programs achieved in the recent years and bring the malaria epidemics to the pre-ACT era. Hence, discovery of new malaria intervention strategies is one of the highest research priorities for the future. For this purpose, understanding of unique biological processes that are essential for malaria parasites growth and development is crucial.

Calcium signaling in* Plasmodium falciparum*, the most dangerous species of the malaria parasites, may represent one such area for drug target explorations. Previous studies established that* P. falciparum* parasites utilize calcium signaling during their life cycle progression. This is demonstrated by the wide spectrum of genes encoding calcium-dependent protein kinases and calmodulins present in the* P. falciparum* genome [5–8] but also by the tightly regulated cytoplasmic calcium concentration via intracellular calcium stores [9]. Calcium has been shown to be important for the parasite maturation [10, 11] and for the vital parasitic processes such as invasion, gliding motility [11–18], and sexual stage development [19–22]. Nonetheless, many gaps remain in the comprehensive understanding of the role of calcium signaling in* Plasmodium* parasites. In particular, very little is known about the role of calcium signaling in transcriptional regulation of* P. falciparum.* Given that in other eukaryotic cell systems calcium-dependent transcription has a wide range of biological functions [23–28], it is reasonable to expect that in* Plasmodium*, calcium-dependent intracellular signaling is also linked with transcription, regulating multiple mechanisms important for the parasite growth, development, and adaptation to its host environment.

One of the most widely used tools for studying calcium signaling in eukaryotic cells is calcium ionophores that are able to abolish electric potential and Ca^2+^ gradients maintained at intracellular membranes, thereby mobilizing intracellular calcium stores. There are numerous examples where calcium ionophores have been used to explore calcium signaling events in eukaryotes including transcription. Genes encoding glucose-regulated proteins in hamster fibroblasts have been shown to be induced by A23187 mediated depletion of intracellular calcium stores [29]. It was also shown that an increase in the cytosolic calcium concentration induced by A23187 and ionomycin was able to trigger the commitment to differentiation and increased expression of erythroid genes in murine erythroleukemia cells [30]. Ionomycin was also shown to be able to induce calcium flux and thus expression of the T-cell CD7 gene [31]. The changes in the intracellular calcium concentration induced by calcium ionophores is also able to alter the induction of p33 gene expression by insulin [32]. There is strong experimental evidence that ionophore compounds can affect the internal calcium stores in* Plasmodium* parasites. First, calcium-imaging studies of* P. berghei* using fluorescent calcium indicators demonstrated that ionomycin increases cytoplasmic calcium concentrations from a nonacidic calcium-rich compartment and the alkalinized acidocalcisomes [33]. In* P. falciparum*, ionomycin has been shown to increase the cytoplasmic calcium concentration by releasing calcium from the intracellular calcium stores that include the parasitophorous vacuole (PV) [5, 34]. Given the high activity of the calcium ionophores in the* Plasmodium* cells [35], these compounds provide a suitable tool for studies of calcium-dependent transcriptional processes during the malaria parasite development.

In this study we analyzed the transcriptional responses of* P. falciparum* parasites to two calcium ionophores, ionomycin and A23187, in order to evaluate the effect of changing calcium distribution within the cell. We show that both inhibitors induce overlapping but not identical changes of the* P. falciparum* transcriptome ranging from up- and downregulation of many genes of a narrow set of biochemical and cellular pathways (for ionomycin) to overall developmental arrests (for A23187). Both inhibitors, however, cause a strong inhibition of transcriptional activity of essentially all genes of the apicoplast genome. Focusing on this phenomenon, we identified a nuclear encoded apicoplast targeted protein (MAL13P1.156) that carries a calcium binding (EF hand) domain. Overexpression of MAL13P1.156 confers an increase in the resistance of* P. falciparum* parasites to ionomycin, which suggests that this protein might play a role in calcium-dependent signaling pathway(s) in the apicoplast.

## 2. Results

### 2.1. Transcriptional Responses of* P. falciparum* to Calcium Ionophores

**Figure 1:**
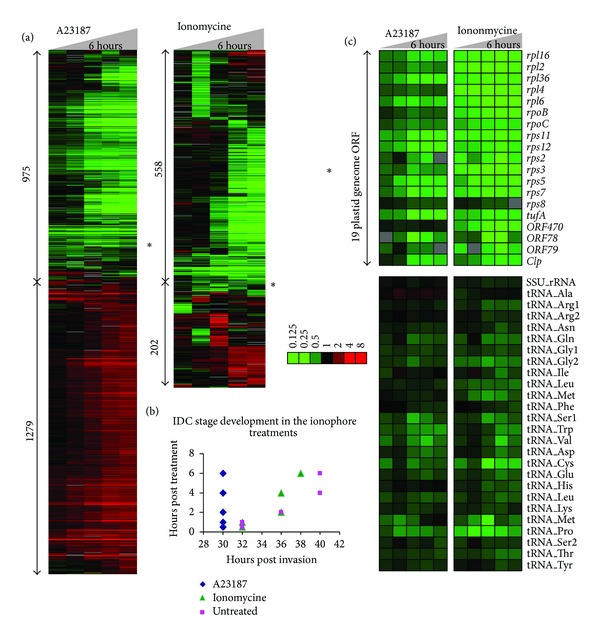
Transcriptional response of* P. falciparum *schizonts to calcium ionophores, ionomycin, and A23187. (a) Total 975 and 558 genes were downregulated and 1279 and 202 upregulated by >2-fold by A23187 and ionomycin, respectively. The heatmaps show relative mRNA levels in each time point compared to the corresponding time point in the untreated cells. The color code corresponds to log⁡⁡2 ratios mRNA abundance between the treatment and untreated controls. The vast majority of the genes show a gradual change throughout the 6-hour treatment time courses (materials and methods) with the exception of a small gene cluster that showed much rapid decrease in mRNA abundance in both inhibitor treatments (∗). (b) Pearson correlation between the treatment time points and the reference IDC transcriptome revealed that A23187 caused a developmental arrest of the* P. falciparum* development at 30 hours after invasion (hpi) (early schizont stage). In contrast, the ionomycin treated parasites exhibit expected progress through 6-hour treatment, from 32 to 38 hpi, hence indicating no developmental arrest by this inhibitor. (c) The heatmap shows relative mRNA expression level of the apicoplast genome coding genes.

In the first step we wished to investigate genome-wide gene expression responses of* P. falciparum* parasites to ionomycin and A23187, two calcium ionophore compounds whose effect (presumably) leads to a release of the internal calcium stores and thus altering Ca^2+^ concentration in essentially all intracellular compartments. For this we carry out 6-hour treatments of highly synchronized* P. falciparum* culture at the schizont stage (~30 hours after invasion, hpi) with 5 *μ*M of both ionophores (Dataset S1, available online at http://dx.doi.org/10.1155/2014/869401). Subsequent genome-wide gene expression analysis (see materials and methods) revealed that both compounds induced significant transcriptional responses (Figure 1(a)). Intriguingly, in spite of the fact that both ionomycin and A23817 have an identical activity, transport of Ca^2+^ across the membranes of eukaryotic cell compartments, the effect of these inhibitors on the* P. falciparum* transcriptome was not identical. The exposure of the* P. falciparum* parasites to A23817 led to differential expression of at least 2254 genes (1279 up- and 975 downregulated genes by >2-fold). As previously demonstrated, such broad transcriptional changes are typically not the reflections of specific transcriptional responses but rather a result of a developmental arrest [36, 37]. To test this possibility, we utilized a recently developed algorithm that allows developmental stage evaluation (“IDC aging”) by assessing the correlation of the entire expression profiles in each experimental time point to the high resolution* P. falciparum* IDC transcriptome [38]. Indeed, all five time points of the A23817 treatments have mapped to the 30 hpi which corresponds to the early schizont stage that was used as a starting point of all treatment experiments (Figure 1(b)). Hence the majority of the A23817-induced differential expression corresponds to the mRNA differences between the starting/arrested parasite cultures compared to the untreated controls that progressed through the mid-to-late schizont stage normally (Figure 1(b)). In contrast to A23817, ionomycin did not arrest the* Plasmodium* schizont stage progression and the global transcriptional profiles of the five treatment time points mapped to the expected IDC timing (32–38 hpi). Instead, this compound induced a specific transcriptional response with 202 genes up- and 558 genes downregulated. Interestingly, the group of upregulated genes was statistically enriched for factors of host parasite interaction including a subgroup of the* var* gene family, the main antigenic determinants during* P. falciparum* infection. In contrast, the group of downregulated genes contained large number of factors of merozoite invasion including merozoite surface antigens, actomyosine motors, and resident proteins of invasion related organelles, rhoptry, microneme and dense granules (data not shown). In addition, 21 out of total 55 genes were predicted to play a role in the fatty acid synthesis in the apicoplast (as annotated by the Malaria Parasite Metabolic Pathway database [39]). This represents a strong statistical enrichment (*P* value ~ 0) and indicates a specific effect of ionomycin on the function of the apicoplast.

Corroborating this observation, both A23817 and ionomycin caused a dramatic downregulation of the vast majority of genes encoded by the plastid (apicoplast) genome. This is demonstrated by a unique narrow gene cluster with tightly correlated expression profiles (Pearson correlation > 0.93) that are characterized by a dramatic decrease of mRNA levels even in the early time points of the treatment (0.5 and 1 hour after treatment) (Figure 1(a), asterix). This is in sharp contrast with the majority of the ionophore-induced transcriptional differences that are gradual (Figure 1(a)). Visual inspections of these narrow gene clusters revealed a strong overrepresentation of plastid genome encoding genes. Total 19 out of the 29 plastid genes represented on the microarray were found downregulated as a result of both ionophore treatments (Figure 1(c)) (10 remaining ORFs showed no expression). In addition, all plastid tRNAs (25) and one rRNA genes that were detected by the microarray analysis also show decreased levels in the ionophore treatments compared to the untreated cells (Figure 1(c)). Moreover, two apicoplast encoded genes of the subunits of the putative apicoplast RNA polymerase (*rpoB* and* rpoC*) were downregulated by both ionophores. This is in contrast to the third subunit (*rpoA*) which is encoded by the nuclear genome (PF3D7_1307600) whose expression is unaffected by neither of the inhibitors (data not shown). This suggests that the Ca-dependent regulation of the apicoplast gene transcription is independent of the nuclear genome.

Taken together, A23817 and ionomycin have a profound effect on the* P. falciparum* transcriptional cascade with the former causing a developmental arrest and the latter inducing specific transcriptional changes of the nuclear encoded genes. Besides this, both compounds have exhibited a strong inhibitory effect on the plastid transcription, downregulating essentially all genes encoded by the 35 kb DNA genome of this organelle.

### 2.2. Calcium Ionophores Neither Inhibit Apicoplast DNA Replication Nor Interfere with the Normal Apicoplast Development

**Figure 2:**
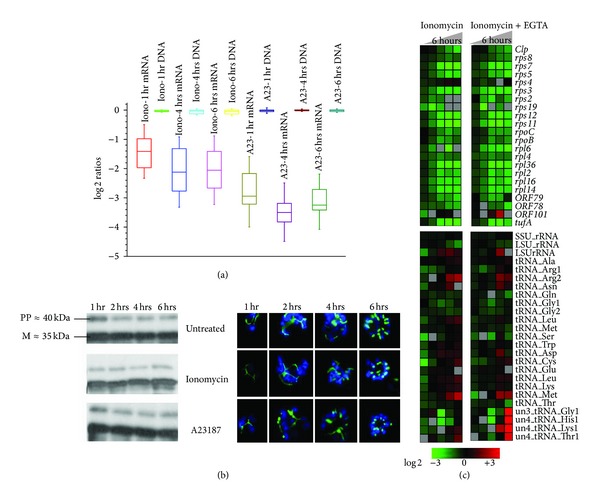
Apicoplast development and DNA replication are hardly inhibited by calcium ionophores. (a) Apicoplast DNA replication is not inhibited by calcium ionophores. Box plots for the log⁡⁡2 expression ratios of averaged oligos representing all apicoplast genes (obtained from the microarray hybridization results filtered for 3-fold change in 2 time points) and log⁡⁡2 ratios of the treated and untreated apicoplast DNA (obtained from a CGH experiment where the total DNA from the treated parasites was hybridized against the total DNA from the untreated parasites) have been compared. (b) Western blots show the processed band (≈35 kDa) of the ACP-GFP fusion protein (≈40 kDa) directed towards apicoplast indicating that the protein has been imported and processed without much interference even after 6 hours after treatment. Fluorescence microscopy done on ACP-GFP expressing parasites confirms the western results. (c) Relative expression of apicoplast genes in ionomycin treatment of schizonts both in presence and absence of 3 mM EGTA. (I-ionomycin, I + E-ionomycin plus EGTA).

Given the consistency of the ionophore-mediated downregulation of the apicoplast genes, we hypothesize that transcriptional regulation in the* P. falciparum* plastid is sensitive to Ca^2+^ concentration. To support this model, we wished to exclude the possibility that the decreased mRNA levels of the plastid genes are a simple reflection of discrepancies in the apicoplast development, particularly apicoplast DNA replication that is rapidly ongoing during the schizont stage. Hence we carried out comparative genomic hybridization (CGH) with total DNA isolated from schizonts treated by both calcium ionophores (Figure 2(a), Dataset S2). Even after 6 hours of treatment with both inhibitors, the apicoplast DNA content was not affected while the transcription was repeatedly downregulated as seen in the initial transcriptome analyses (Figure 2(a)). Interestingly, A23187 has much stronger effect on the mRNA level of the apicoplast genes reducing their content by median 3.24 ± 0.12 compared to ionomycin that caused reduction by 2.05 ± 0.15 after six hours of treatment.

According to previous studies on plant cells, calcium ionophores are able to inhibit protein import into the chloroplast, the orthologous endosymbiotic organelle of the apicoplast. Import of proteins with a cleavable signal peptide into the isolated intact chloroplasts can be inhibited by calcium ionophores as a consequence of emptying chloroplast calcium content [40]. In* P. falciparum*, 545 nuclear encoded proteinsare predicted to be imported into the apicoplast facilitating numerous metabolic and cellular processes associated with this compartment [41]. Inhibition of the import of these proteins will likely cause major disruptions to the apicoplast morphology as well as function. In order to investigate the effect of the ionophores on apicoplast proteins import, we utilize the acyl carrier protein (PfACP, PFB0385w) as a molecular marker of the apicoplast (Figure 2(b)). PfACP is one of the major apicoplast factors that are implicated in the type II fatty acid biosynthesis [42]. This protein carries an N-terminal signal sequence that targets its localization to the apicoplast [43]. For our study, we generate a transgenic* P. falciparum* cell line with PfACP episomal overexpression. Fluorescence microscopy analysis of the pattern of intracellular localizations of the PfACP in both ionomycin and A23187 treated cells is essentially identical to untreated cells. Similarly neither of the ionophores affected the pattern of posttranslational processing of PfACP (Figure 2(b)). The full-length apicoplast targeted proteins undergo processing by a stromal processing peptidase upon the import into the apicoplast as previously described [44]. In our results, the signal peptide cleavage is undisturbed even 6 hours after treatment (Figure 2(b)). Hence we conclude that in neither of the treatments we observe any major interference with the PfACP import into the apicoplast.

Based on previous studies of other eukaryotic systems, the ionophore-induced increases of cytosolic calcium concentrations are mediated by two possible mechanisms. These include an influx of calcium from the ambient media via native Ca^2+^ channels and a phospholipase-C (PLC) mediated calcium release from the intracellular calcium stores [45].In the next step, we wished to test whether the ionophore effect on the apicoplast transcription is mediated by a general influx of Ca^2+^ from the extracellular medium into the parasite cytoplasm and subsequently to the apicoplast or whether it is associated with the redistribution of intracellular calcium concentration within the parasite cell. In a previous study* Trypanosoma cruzi *the causative agent of Chagas disease, it was shown that EGTA can be used to chelate the extracellular calcium, eliminating it of its influx into the ionophore treated cells and the majority of the phenotypic effect induced by the ionophores is due to redistribution of the intracellular of calcium [46]. Similar to these studies, we carried out additional transcriptome analyses where the parasites were treated with 5 *μ*M ionomycin and at the same time the extracellular calcium was depleted by chelation with 3 mM of EGTA. As expected, we observed a similar effect of the downregulation of apicoplast gene transcription that occurs to the same degree compared to cells grown in the calcium presence (Figure 2(c), Dataset S3). These results suggest that the redistribution of the intracellular calcium such as release of the intracellular calcium stores and not the extracellular calcium influx is responsible for the observed ionophore effect on apicoplast transcription.

Taken together, these experiments show that neither of the ionophores caused dramatic disruptions of the apicoplast DNA replication or protein import. In addition, the microscopy studies did not detect any major abnormalities in the apicoplast morphology during its growth and division in the late schizont stages. Although the used techniques could not exclude subtle changes in the apicoplast morphology or protein content, these data suggest that the observed dramatic reduction of the apicoplast gene mRNA levels indeed represents a reduced transcriptional activity at the apicoplast genome. Moreover, the ionophore-induced transcriptional changes in the apicoplast are associated mainly (if not fully) with the intracellular stores of Ca^2+^. Hence, the apicoplast transcriptional regulation is sensitive to fluctuations of Ca^2+^ concentration likely within the apicoplast itself.

### 2.3. Calcium Signaling in the Apicoplast

**Figure 3:**
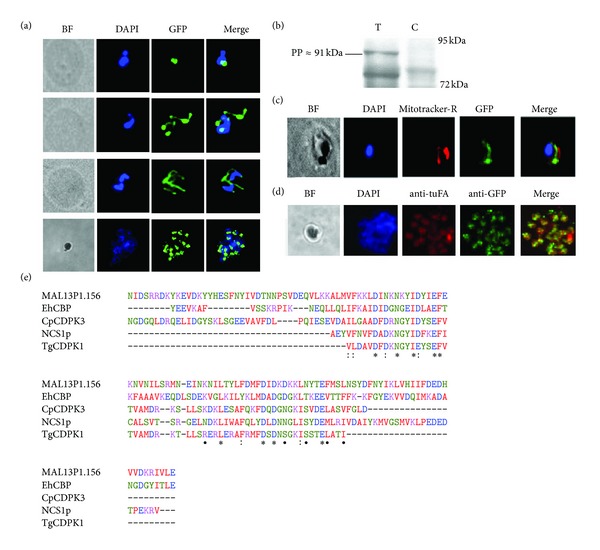
Identification of an apicoplast targeted protein with EF-hand domain. (a) Immunofluorescence microscopy done (anti-GFP) on parasites episomally expressing MAL13P1.156-GFP fusion shows typical apicoplast pattern. (b) Antibody against the c-terminal GFP detects MAL13P1.156 full-length protein with an apicoplast targeted protein signature (T-transfected, C-Control). (c, d) Colocalisation with Mitotracker-Red and apicoplast encoded tuF, respectively. (e) MAL13P1.156 multiple alignments with the structural homologues obtained from a sequence blast on PDB. EhCBP-*Entamoeba histolytica* calcium binding protein, CpCDPK3-*Cryptosporidium parvum* calcium-dependent protein kinase3, NCS1p-calcium binding protein NCS-1* Schizosaccharomyces pombe*, and TgCDPK1-*Toxoplasma gondii* calcium dependent kinase1.

Given the potential role of calcium on transcription of the apicoplast genes, we wished to identify a protein factor(s) that may facilitate this phenomenon. For this we carried out bioinformatics analyses of all 545 plastid targeted nuclear encoded proteins [47, 48] and identified MAL13P1.156, a calcium binding protein that contain a signal anchor with probability of 0.954 [49] and 5 of 5 positive tests for an apicoplast targeting peptide [47] at its C-terminus. A similar protein that lacks an apparent signal peptide but contains a signal anchor has been found to be targeted to the apicoplast via an independent bipartite signal targeting in* Toxoplasma gondii* [50, 51]. MAL13P1.156 is a single exon gene (1599 bp) that codes for a 64 kDa protein and contains an EF-hand domain (prediction* e*-value, 2.80*E* ^−13^) at the position between 234 and 518 amino acid of the deduced polypeptide. A search of the RCSB Protein Data Bank (PDB) [52] for structurally similar proteins retrieved several sequences of calcium binding proteins with the EF-hand calcium binding domain. These include an EF-hand calcium binding protein from* Entamoeba histolytica* (*E*-value 0.002, solution NMR), CDPK3 (calcium-dependent protein kinase-3) from* Cryptosporidium parvum* (*E*-value 0.053, X-ray diffraction), myristoylated NCS1p from* Schizosaccharomyces pombe* (*E*-value 0.10, solution NMR) and CDPK-1 (calcium-dependent protein kinase-1) from* Toxoplasma gondii* (*E*-value 0.35, X-ray diffraction). Multiple alignments of the amino acids spanning the EF-hand domain (214th to 341th positions) show considerable conservation of the calcium binding domain between these structural homologues (Figure 3(e)). This suggests a putative calcium binding function of MAL13P1.156 and thus its role in calcium-dependent signaling in the apicoplast such as sensing and/or buffering free Ca^2+^ ions similar to its plant counterparts in the chloroplast [53].

To investigate the biological function of MAL13P1.156, we constructed a fusion construct of this protein with a C-terminal GFP and subsequently generated a* P. falciparum* (3D7) transgenic cell line where this fusion protein is expressed episomally. Western blot analyses show a strong expression of this protein as a full-length fusion protein of 91 kDa (64 kDa full-length protein plus 27 kDa GFP) and a processed form ~72 kDa (Figure 3(b)). This proteolytic cleavage is consistent with the signal peptide processing upon import to the apicoplast. Subsequently, immunofluorescence microscopy (IFA) of the transgenic cell line using the anti-GFP antibody shows the characteristic pattern of apicoplast localization with a single small compartment in the early schizonts, elongated branched formation in the late schizonts, and finally divided punctuate formations corresponding to new apicoplast precursors in the newly formed daughter merozoites (Figure 3(a)). Finally, IFA-based colocalization studies revealed a close proximity between the MAL13P1.156 labeled compartments and mitochondria labeled by mitotracker Red (Figure 3(c)). This is consistent with physical association of apicoplast and mitochondria in* P. falciparum*. Finally there is partial but significant colocalization between MAL13P1.156 and the apicoplast marker EF-Tu (Figure 3(d)). Based on these results we conclude that MAL13P1.156 localizes to the apicoplast and hence name this protein* P. falciparum* apicoplast calcium binding protein 1 (PfACBP1).

**Figure 4:**
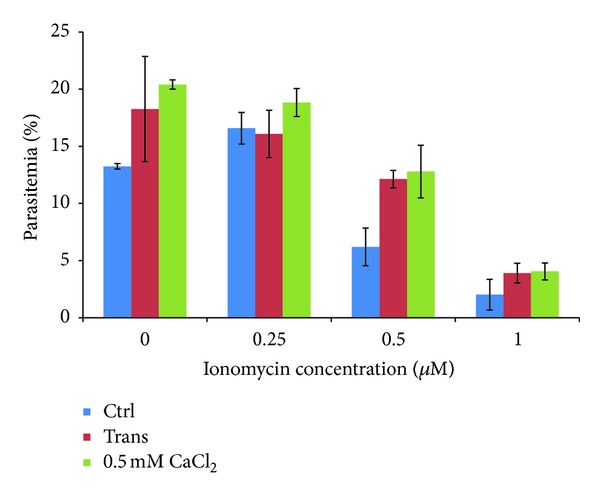
Reduced sensitivity of the transfected cells towards ionomycin. Blue-nontransfected (Ctrl), red-transfected (Trans) and green-nontransfected parasites grown in medium supplied with external calcium (500 *μ*M). *P value: 0.01.

To investigate whether PfACBP1 plays a role in intracellular calcium homeostasis of the* Plasmodium* parasites we determined the sensitivity of the generated transgenic cell line to ionomycin. Here we hypothesize that MAL13P1.156 overexpressing parasites have a higher capacity to withstand ionomycin exposure and its effect as an intracellular calcium ion mobilizer that is presumably depleting the apicoplast of calcium. Overexpression of PfACBP1 would help to retain higher calcium concentration in the apicoplast via its calcium binding properties. To investigate this hypothesis,* P. falciparum* cells (mid schizont stage) were treated with 0, 0.25, 0.5 and 1 *μ*M of Ionomycin for 12–14 hrs until next invasion and the parasite survival was monitored by Giemsa smear microscopy. At 0.5 *μ*M of ionomycin (that roughly corresponds to the ionomycin IC50; see below), the survival of the PfACBP1 overexpressing cell line is ~2-fold higher (*P* value 0.01) compared to the nontransfected parasites (Figure 4). The survival rate of the transgenic cell line is comparable to the nontransfected parasites grown in the medium supplemented with 500 *μ*M of CaCl_2_. Here we assume that the extracellular calcium supplied in the growth medium reduces the severity of the calcium mobilization action of ionomycin (Figure 4). This increase in the resistance of the PfACBP1 overexpressing parasites suggests its role in the calcium signaling in the plastid but also the fact that the calcium depletion from the apicoplast is a part to the toxic effect of ionophores in the* P. falciparum* parasites.

## 3. Discussion

Here we used transcriptional profiling to analyze the mode of action of ionophores (ionomycin and A23187) in order to gain more insights into the role of Ca^2+^ signaling in* P. falciparum*. Although transcriptional profiling is known to be a powerful method to understand activities of small-molecule inhibitors in eukaryotic cells, in* P. falciparum* as well as other highly specialized pathogens, chemical or other types of external stimuli/perturbations do not always induce specific responses [37, 54, 55]. In* P. falciparum*, transcriptional responses to external perturbations range from low amplitude nonspecific changes in mRNA profiles to broad extensive transcriptional changes affecting the vast majority of the* P. falciparum* genes that are typically consistent with developmental arrests and/or induction of the sexual stages. Nonetheless, for several types of small molecule inhibitors, transcriptional responses are more specific, involving genes of direct or indirect targets [56, 57]. In this study, we used two inhibitors that are known to have similar (or overlapping) effects on calcium distribution in the eukaryotic cell. Interestingly these two inhibitors exhibit dramatically different effects on the* P. falciparum* growth. While ionomycin affected expression of a narrow group of genes, with downregulation of plastid gene expression being most pronounced, A23187 caused a broad transcriptional shift that is consistent with a developmental arrest in the schizont stage. This is surprising given that both inhibitors have similar growth inhibitory effects on the* P. falciparum* cells with IC50 (inhibition concentration by 50%) 513 and 304 nM for ionomycin and A23187, respectively (data not shown). This could be either due to a stronger effect of A23187 on calcium redistribution in the cell and subsequently a more dramatic response manifested by the developmental arrest, or alternatively this discrepancy could be caused by A23187 interacting with additional molecular targets compared to ionomycin. Although more research is required to understand the molecular mechanism(s) that underlines the developmental arrests induced by various perturbations, these data further underline the overall diversity of* P. falciparum* transcriptional responses to external stimuli and their utility for systems biology approaches using the “guilty-by-association” principle [56, 58, 59].

The main activity of calcium ionophores is to carry Ca^2+^ cations across the membranes down its concentration gradient [45]. In eukaryotic cells this causes cytoplasmic mobilizations of Ca^2+^ that are released from the intracellular calcium stores, intracellular compartments in which calcium is sequestered under normal growth concentrations. Similar to other eukaryotic cells, the main intracellular calcium stores of apicomplexan parasites include the endoplasmic reticulum (ER), nuclei, and mitochondria as well as specialized acidic compartments, acidocalcisomes [60]. In addition to these canonical calcium stores, the parasitophorous vacuole (PV) (the lumen between the parasite plasma membrane (PPM) and the parasitophorous vacuolar membrane (PVM) diving the parasite cell from the host cell cytoplasm during their intraerythrocytic development) was shown to contain high concentrations of calcium [34]. It was proposed that the high concentration of calcium in the PV, estimated at ~40 *μ*M, creates a calcium rich microenvironment that is essential for the parasite growth in the otherwise calcium poor erythrocyte cytoplasm, >100 nM. Expectedly, ionomycin causes a rapid efflux of calcium out of the PV into the parasite cytoplasm and to the ambient media and thus diminishing the concentration gradient at the parasite plasma membrane. In addition to the PV, ionomycin can also mobilize other intracellular calcium stores in the parasites (presumably ER, mitochondria, and acidocalcisomes) raising the calcium concentration in the parasite cytoplasm even further [34]. The rapid effect on the plastid genome transcription observed in our study suggests that the ionophores can affect the concentration of calcium diverting it from a steady state concentration that is likely essential for the proper function of this organelle. This model is supported by the fact that the ionomycin-mediated inhibition of the plastid transcription also occurs when the calcium is chelated from the ambient media (Figure 2(c)); hence, the ionophore-mediated calcium flow is directed away from the parasitized erythrocyte. Moreover, thapsigargin (THG) that increases cytoplasmic calcium by a specific release from the ER [34] has no effect on the plastid genome transcription (data not shown). Taken together these data suggest that in addition to calcisomes, ER, and mitochondria, the plastid can serves as another component of the internal calcium stores in the* Plasmodium* parasites.

There is mounting evidence that calcium signaling plays a major role in maintenance and genesis of the endosymbiotic organelles of eukaryotic cells. In the chloroplast of the plant cells, calcium is an essential signaling factor for at least three different functionalities: import of nuclear encoded proteins, vesicular transport system, and oxygenate photosynthesis (reviewed in [53]). Most of the calcium-dependent signal transduction in the chloroplast is believed to be facilitated by proteins that contain the “EF-hand” domain(s) [61]. Binding of calcium causes conformational changes to the EF-hand proteins that subsequently results in increasing binding affinity to other interacting proteins or DNA sequences. Alternatively, calcium binding could cause cross-activation of enzymatic activities of additional domains present at the polypeptide such as protein kinases in calmodulins, calcium-dependent protein kinases present in plants, and protists [62]. Here we identify a novel EF-hand protein in* P. falciparum*, PfACBP1, that is targeted to the plastid and its overexpression increases resistance of the parasite cells to ionomycin. In our bioinformatics analyses of the 545 plastid targeted proteins [47, 48], PfACBP1 showed the highest homology to the EF-hand consensus sequence which suggests its crucial role in calcium-mediated regulatory function(s) in this compartment. In future studies it will be intriguing to explore the role of PfACBP1, the highly conserved calcium binding protein, in regulation of plastid gene expression.

Until today, the plastid represents one of the most important targets for malaria chemotherapy [63]. A number of apicoplast functionalities can be targeted by specific well-established antimicrobial chemotherapeutics, some of which can be used for malaria treatment and prophylaxis. These chemotherapeutic strategies take advantage of the prokaryotic character of several basic apicoplast mechanisms including DNA replication, inhibited by fluoroquinolinone antibiotics [64]; RNA transcription inhibited by rifampicin [65]; and protein translation that can be blocked by clindamycin, azithromycin binding to 23S rRNA [66], and doxycycline and tetracycline binding to 16S rRNA [67]. All these compounds were shown to block apicoplast organellogenesis and division which leads to an absence of this compartment in the newly invaded parasite generations. Although these parasites could develop until the midstage of the (subsequent) IDC, the lack of apicoplast functionalities likely causes the ultimate cell death. Overall, this phenomenon, also known as the delayed death phenotype, is characteristic for most of these drugs with the exception of tetracycline that is believed to also affect the mitochondrion and kill the cells instantly [67]. In addition to rifampicin and other RNA synthesis blockers, doxycycline was also found to specifically and exclusively inhibit transcription of the apicoplast genome encoded genes [68]. This is somewhat surprising as the main mode of action doxycycline is blocking the apicoplast proteosynthesis. This suggests that transcriptional regulation of the apicoplast genes involves multiple components of intracellular signaling potentiality including apicoplast encoded proteins. Here we show that calcium-dependent signaling factors contribute to this regulation and that interference with these can also have toxic effect on the* Plasmodium* cells, albeit not via the delayed death mechanism.

Several biochemical pathways associated with the apicoplast are being explored as suitable drug targets for malaria chemotherapy. These include fatty acid type II (FASII) [69], nonmevalonate isoprenoid synthesis [70], apicoplast REDOX system [71], and heme synthesis [72]. Each of these pathways represents essential biological processes that take place within the endosymbiotic organelle and thus were retained through the evolution. With that, each pathway retained a certain portion of prokaryotic features that are being explored by malaria drug development efforts. The most remarkable example represents FASII that arrears to be the sole producer of fatty acids in* Plasmodium* cells as precursors of membrane synthesis and energy stores. Several inhibitors of FASII enzymes are being explored as suitable drug candidates including trichlosan (inhibitor of enoyl-ACP reductase, FabI) and thiolactomycine (inhibitor of beta-ketoacyl-ACP synthetase II and III, Fab II and III) (reviewed in [63]). Although the validity of this pathway as a drug target for blood stage parasites has been recently disputed by observations that apicoplast plays only a minor biochemical role during its asexual intraerythrocytic development [73]. FASII was found to be predominant and extremely important during the* Plasmodium* liver stage development [74]. Indeed, the* Plasmodium* liver stages appear to be highly sensitive to a FASII inhibitor hexachlorophene, as well as to rifampicin and tetracycline [74]. Given the importance of calcium-dependent signaling in the apicoplast transcription it will be interesting to explore its potential as a new target for liver stage drug development which is one of the main objectives of the future programs for malaria control and elimination proposed for the next era of malaria-related research [75].

## 4. Materials and Methods

### 4.1. Cell Culture, Drug Treatment, and DNA Microarray

All treatment experiments were carried out with the* P. falciparum *3D7 strain. Calcium ionophore treatments were carried out as follows: highly synchronized* P. falciparum* cultures were treated with 5 *μ*M of calcium ionophores, ionomycin and A23187 (Sigma) at the schizont stage for 30 minutes, 1 hour, 2 hours, 4 hours, and 6 hours. Total RNA from each of the time points was isolated and aminoallyl-cDNA was synthesized using reverse transcriptase system (Fermentas). Subsequently cDNA made from the treated and untreated parasites were labeled with Cy5 (GE-Amersham). A reference pool was made by mixing equal amount of RNA from the parasites collected at 6 hours interval throughout the 48 hours life cycle and was labeled with Cy3 (GE-Amersham). The samples were then hybridized on a spotted cDNA chip platform comprising 10166 MOEs representing 5363 coding sequences [36]. The data was normalized and filtered with the condition, signal intensity > background intensity + 2 SD of background intensity using NOMAD (http://derisilab.ucsf.edu). Hierarchical clustering of the log-transformed ratios was then done using Cluster (Eisen lab) [76] and visualized using Treeview (Eisen lab) [76]. Pathway analysis was done based on the hyper geometric and binomial probability distribution and pathways which had a* P* value of <0.01 were considered significant. For the extracellular calcium chelation experiment, the medium was treated with 3 mM of EGTA and the ionomycin treatment was carried out later on at 5 *μ*M concentration. Hybridization and data analysis were carried out as above.

### 4.2. Comparative Genomic Hybridization (CGH)

Comparative genomic hybridization was carried out with total DNA isolated from the untreated parasites and the parasites treated with the calcium ionophores (ionomycin and A23187 at 5 *μ*M concentration) at 1 hour, 4 hours, and 6 hours after treatment.

3 *μ*g of the total DNA from each of the samples was subjected to klenow (NEB) reaction as described before [77]. Treated DNA labeled with Cy3 was then hybridized against untreated DNA labeled with Cy5. Hybridization and Data analysis were done as described above for the cDNA hybridization.

### 4.3. Transfection

Transfection of 3D7 parasites was performed as described before [78]. Two lines of transgenic parasites episomally expressing GFP fused to the C-terminus of acyl carrier protein (ACP) and MAL13P1.156, respectively, were developed. ACP and MAL13P1.156 were amplified from the 3D7 genomic DNA using the following primers:

 ACP XhoI-Fw
 5′-AGTC**CTCGAG**CACCTTATTAGAATGAAGATCTTATTACTTTG-3′
 ACP-AvrII-Bw
 5′-AGTC**CCTAGG**TTTTAAAGAGCTAGATGGG-3′
 MAL13P1.156 XhoI-Fw
 5′-AGTC**CTCGAG**ATGAAACTTTTAAATTTTCCACTGTCC-3′
 MAL13P1.156 AvrII-Bw
 5′-AGTC**CCTAGG**TGTGGCATATACTATGTCTGGAGCC-3′.

pARL vector [79] was modified for generating the required constructs. Stevor gene was replaced with ACP and MAL13P1.156 for generating pARL-ACP-GFP and pARL-MAL13P1.156, respectively, under the control of PfCRT promoter and hDHFR as the selectable marker.

### 4.4. Fluorescence Microscopy

Parasites expressing ACP-GFP episomally was used for the import inhibition experiment. Smears were made from parasites exposed to ionomycin and A23187 1 hour, 2 hours, 4 hours, and 6 hours after invasion. The smears were fixed in 4% paraformaldehyde and, after staining with DAPI, were viewed under a fluorescence microscope. For the localization and colocalization experiments, immunofluorescence assay was done using anti-GFP (mouse anti-GFP, Santacruz Biotech) and anti-tuFA (rabbit anti-tuFA, kindly given by Dr. Saman Habib, CDRI, India).

### 4.5. Western Blots

Proteins from the crude parasite lysates were separated on a 10% SDS polyacrylamide gel. The resolved proteins were then transferred to a nitrocellulose membrane. The blots were then probed with rabbit anti-GFP antibody which was in turn detected with anti-rabbit IgG conjugated with HRP.

### 4.6. Drug Assay

Nontransfectants, nontransfectants with 500 *μ*M CaCl_2_ in the medium, and the transfectants overexpressing MAL13P1.156 were exposed to ionomycin at 0, 0.25, 0.5, and 1 *μ*M concentrations in the schizont stage. The newly invaded rings were counted on giemsa stained smears under a light microscope. The percentage of rings was plotted against the inhibitory concentrations.

## Supplementary Material

The supplementary material contains three dataset representing the three featured microarray based genomic studies: Dataset 1 (Transcriptional responses to Ionomycine DS1). This table contains results from the analysis of genome-wide gene expression responses of *P. falciparum* to Ionomycine and to A23187 as described in Figure 1. Briefly, P. falciparum cultures were treated by both compounds for 30 min, 1.0, 2.0, 4.0 and 6.0 hours and the cell samples were collected for total RNA isolations from both the treatment experimental courses (Inono-30 mins, Iono-1 h, e.t.c.) and untreated control cultures (ctrl-30mins, e.t.c.). The table summarizes relative mRNA levels of all P. falciparum genes determined by two channel competitive microarray hybridization were each sample was hybridized against an arbitrary RNA pool (for more details see materials and methods). Dataset 2 (CGH-DS2). This table contains results from microarray based comparative genomic hybridization (CGH) measuring the copy number of each gene in all time points from the ionophore treatment time courses as described for Dataset 1. The final findings from these the CGH experiment is featured in Figure 2 (a). Dataset 3 (Schizonts_IonomycineEGTA_DS3). This table summarizes results from genome-wide gene expression analyses of *P. falciparum* response to Inonomycine in the presence and absence of external calcium chelator EGTA. Briefly, the cells were treated for 30 min, 1.0, 2.0, 4.0 and 6.0 hours with Inonomycine of with Inonomycine+EGTA. Total RNA was isolated and subjected to a microarray analysis of mRNA expression levels for all *P. falciparum* genes using CGH as described in materials and methods. The labels of the experimental time points are analogous to the dataset 1. The findings from these experiments are described in Figure 2 (c).
